# Whole-Genome Sequence of a *Megasphaera elsdenii* Strain Isolated from the Gut of a Healthy Indian Adult Subject

**DOI:** 10.1128/genomeA.01033-17

**Published:** 2017-10-19

**Authors:** Satyabrata Bag, Tarini Shankar Ghosh, Bhabatosh Das

**Affiliations:** Molecular Genetics Laboratory, Centre for Human Microbial Ecology, Translational Health Science and Technology Institute, NCR Biotech Science Cluster, Faridabad, India

## Abstract

*Megasphaera elsdenii* has been previously reported in the gut of ruminating animals. Its role as an animal probiotic is being investigated, specifically from the perspective of enhancing animal productivity. Herein, we report the draft genome sequence of *M. elsdenii* strain indica isolated from the stool sample of a healthy Indian subject.

## GENOME ANNOUNCEMENT

*Megasphaera elsdenii*, traditionally reported in the gut of ruminating animals, is being explored as a probiotic in animal husbandry, specifically for enhancing animal productivity (1–3). However, several studies have indicated both beneficial and detrimental associations of *Megasphaera* species with the health status of the host (1–4). Studies on human gut microbiota have revealed its association with obesity (4). The genus *Megasphaera* is widely distributed in the gut of healthy Indians living across the country (5).

In the present study, *M. elsdenii* strain indica was isolated from the fecal sample of a healthy Indian adult subject. The fresh fecal sample was immediately resuspended in prereduced phosphate-buffered saline (PBS) and subjected to serial dilution. Diluted samples of approximately 100 μl were plated onto tryptic soy agar (Difco) supplemented with 5% (vol/vol) defibrinated sheep blood. The plates were subsequently incubated in an anaerobic workstation (Whitley DG250) filled with an anaerobic gas mixture (80% N_2_-10% CO_2_-10% H_2_) for 24 h at 37°C.

Genomic DNA of *M. elsdenii* strain indica was extracted using the THSTI method (6). Approximately 1 µg of genomic DNA was used for constructing a whole-genome shotgun library. For this purpose, we first performed nebulization of the genomic DNA at 15 lb/in^2^ for 60 s to generate DNA fragments (average size 1.6 kb). DNA fragments were then end polished using T4 polymerase, polynucleotide kinase, and *Taq* polymerase (Roche, USA). These end-polished DNA fragments were then ligated with sequencing adaptor (Roche). The genomic libraries were purified using AMPure XP beads (Beckman Coulter, Inc., USA). The quality of the DNA library was tested using a high-sensitivity DNA chip compatible with the 2100 Bioanalyzer (Agilent, USA). Library quantitation was done using PicoGreen dye in a QubitFluorometer (Invitrogen, USA). Emulsion PCR (Mastercycler proS PCR systems; Eppendorf, Germany) was performed to amplify approximately 7 × 10^7^ DNA molecules per sample. The amplified products were purified using Biomek 3000 (Beckman Coulter, Inc.). Finally, sequencing was performed in a picotiter plate in a 454 GS-FLX+ genome sequencer (Roche).

Whole-genome sequencing of *M. elsdenii* strain indica, followed by sequence assembly using the GS *de novo* assembler, yielded 26 contigs with a total sequence volume of 2,429,033 bp (*N*_50_, 137,903 bp; GC content, 53.2%). Annotation of the draft genome using the Rapid Annotations using Subsystems Technology (RAST) annotation pipeline identified 2,184 coding genes and 75 RNA genes. The genome of *M. elsdenii* strain indica encodes 39 genes conferring resistance to antibiotics and toxic compounds.

### Accession number(s).

The draft genome sequence of *M. elsdenii* strain indica has been deposited at DDBJ/ENA/GenBank under the accession number NQMW00000000. The version described in this paper is version NQMW01000000.
